# Study on the Effect of Temperature on the Self-Healing Behavior of Film Capacitor Dielectrics

**DOI:** 10.3390/ma18174033

**Published:** 2025-08-28

**Authors:** Mengjia Feng, Zhiguo Jia, Yancheng Liu, Yandong Liu, Jia Shi, Chaoyue Zhao, Tianqi Sun, Hongbo Liu, Yunqi Xing

**Affiliations:** 1 State Key Laboratory of Intelligent Power Distribution Equipment and System, Hebei University of Technology, Tianjin 300123, China; 202321401044@stu.hebut.edu.cn (Z.J.); 202321401053@stu.hebut.edu.cn (Y.L.); 202421401116@stu.hebut.edu.cn (Y.L.); 202431402155@stu.hebut.edu.cn (J.S.); 202431402163@stu.hebut.edu.cn (C.Z.); 202421401132@stu.hebut.edu.cn (T.S.); hbliu@hebut.edu.cn (H.L.); yqxing@hebut.edu.cn (Y.X.); 2 School of Electrical Engineering, Hebei University of Technology, Tianjin 300123, China; 3 National Key Laboratory of Electronic Thin Films and Integrated Devices, University of Electronic Science and Technology of China, Chengdu 611731, China; 4 Key Laboratory of Engineering Dielectrics and Its Application, Ministry of Education, Harbin University of Science and Technology, Harbin 150080, China

**Keywords:** self-healing, high temperature, dielectrics

## Abstract

Self-healing is imperative for the restoration of the insulation state of metallized film capacitors following breakdown during operation, thereby ensuring the safe and reliable functioning of the capacitors. Temperature is an important factor affecting the self-healing behavior of film capacitor dielectrics, but the mechanism is currently unclear. To investigate the effects of temperature and dielectric matrix on self-healing behavior, polyetherimide (PEI), cycloolefin copolymer (COC), and biaxially oriented polypropylene (BOPP) were selected as research subjects. A systematic study was conducted to examine the self-healing performance at 80, 120, and 150 °C, as well as the effect of self-healing on insulation and energy storage performance. The results showed that as the temperature increased, the capacitance of PEI decreased by 11.90%, 23.00%, and 37.88%, respectively. COC decreased by 11.76%, 7.63%, and 12.18%, respectively, while BOPP decreased by 8.75% and 9.67%, respectively. The accumulation of breakdown holes formed by self-healing decreases, the area of evaporated electrodes decreases, and the boundaries between the evaporated electrode areas formed by self-healing and the surrounding electrodes become more distinct. Furthermore, COC exhibits high dielectric strength, low dielectric loss, and self-healing properties comparable to BOPP at 150 °C, suggesting significant application potential. This research work is of great reference value for establishing the theoretical relationship between the chemical composition of dielectrics and their self-healing ability, and is of great significance for ensuring the safe and reliable operation of capacitors.

## 1. Introduction

Film capacitors have been shown to exhibit fast charging and discharging speeds, low loss, and excellent stability. This renders them highly promising for applications in the drive systems of new energy electric vehicles, laser pulse weapons, high-voltage flexible direct current transmission, and other fields [1,2,3,4,5]. In the event of a capacitor’s breakdown, the dielectric at the point of breakdown undergoes vaporization due to the immense heat generated. This process results in the evaporation of the electrodes surrounding the breakdown point, creating a high-temperature gas that subsequently evaporates the electrodes. Consequently, the establishment of a conductive path at the breakdown point is impeded, resulting in the capacitor’s reversion to its insulated state. This property is a hallmark of polymer film capacitors, ensuring their long-term stability and reliability [6,7,8].

In the context of polymers with the general formula C_α_H_β_O_γ_N_δ_S_θ_, it has been observed that a high ratio of (α + δ + θ) to (β + γ) is often associated with a diminished capacity for self-healing [9,10]. Presently, commercially available BOPP exhibits the lowest ratio of (α + δ + θ) to (β + γ) among commonly used polymer dielectrics, and its self-healing properties have been the most extensively studied [11,12,13]. Cheng et al. introduced a layer of polymethyl methacrylate (PMMA) film on the outer side of a polypropylene film with the objective of increasing the oxygen content in the composite film. This, in turn, resulted in a reduction in the amount of conductive deposits after self-healing occurred [14]. Li et al. oriented two-dimensional Al_2_O_3_ in BOPP, thereby allowing more of the energy generated during the self-healing process to be used to break down the film. This, in turn, reduced the self-healing area and the degree of capacitance decline [15].

The maximum operating temperature of BOPP is currently only 125 °C [16]. However, the trend toward miniaturization and integration of electrical equipment necessitates dielectric materials capable of operating at higher temperatures [17]. Consequently, researchers have developed aromatic polymers that exhibit excellent thermal stability, including PEI, polyimides (PI), fluorene polyester (FPE), polyethersulfone (PESU), and polycarbonates (PC) [18,19,20,21]. The ratio of (α + δ + θ) to (β + γ) in aromatic polymers is lower than that in BOPP, resulting in poorer self-healing performance. Huang et al. modified the benzene ring structure of PI by substituting it with a carbon ring structure. This alteration resulted in a reduction in electrical conductivity and an increase in hydrogen content within the molecular chain of PI. Consequently, this modification led to the attainment of high discharge energy density and exceptional self-healing performance [22]. In addition, Chen et al. developed a ladderphane copolymer with a ratio of (α + δ + θ) to (β + γ) of 0.97, which self-assembles into highly ordered arrays through π–π stacking interactions, while exhibiting excellent energy storage and self-healing properties [23]. In summary, current researchers rely on altering the ratio of (α + δ + θ) to (β + γ) to ascertain whether self-healing performance has improved based on changes in conductive deposits at self-healing sites, electrode evaporation area, and capacitance. However, the relationship between these variables remains unclear, and the mechanism behind self-healing behavior at high temperatures is not well understood. Consequently, the selection of dielectric materials exhibiting varied temperature resistance properties, the investigation of variables in their self-healing behavior at different temperatures, and the systematic analysis of the relationship between variables will be of paramount importance in elucidating the mechanism of self-healing behavior at elevated temperatures.

Consequently, the present study selected PEI, COC, and BOPP as the subjects of investigation. Initially, the thermal performance of the materials was evaluated through testing. Subsequently, a self-built self-healing performance test platform was utilized to compare the three types of dielectric materials in terms of the number of self-healing occurrences and capacitance decline over 16 h under environmental temperatures of 80 °C, 120 °C, and 150 °C and an applied electric field of 200 MV/m. Next, the morphology and elemental composition of the self-healing site were observed using scanning electron microscopy (SEM) and energy dispersive X-ray spectroscopy (EDS), respectively. The effects of self-healing on breakdown and energy storage performance were measured. The simulation was utilized to replicate the evaporation process of the dielectric and gas products during the self-healing process. This study systematically investigated the effect of temperature on the self-healing properties of PEI, COC, and BOPP, providing important reference values for establishing the theoretical relationship between the chemical composition of composite dielectrics and their self-healing capabilities.

## 2. Materials and Methods

### 2.1. Materials

PEI resin (glass transition temperature [T_g_] = 237 °C) and BOPP film (thickness: 9.8 μm, specifications: 210 mm × 297 mm) were purchased from PolyK Technologies, LLC, State College, PA, USA. COC is the commercially available Topas 6017s-04. N-methylpyrrolidone (NMP, AR, ≥99%[GC], 500 mL) was purchased from Shanghai Aladdin Biochemical Technology Co., Ltd., Shanghai, China. Xylene (99%,Water ≤ 50 ppm [by K.F.], MkSeal) was purchased from Shanghai Macklin Biochemical Technology Co., Ltd., Shanghai, China. All of the above items were used directly.

### 2.2. Sample Preparation

**Figure 1:**
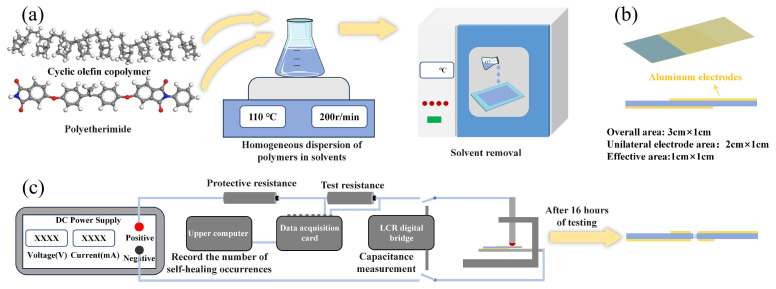
(**a**) Sample preparation process. (**b**) Structure of self-healing performance test samples. (**c**) Self-healing performance test platform.

Preparation of COC film: The primary preparation process is illustrated in Figure 1a. The first step in the procedure was to place 1.2 g of COC in 10 mL of xylene. Then, the mixture was stirred on a magnetic stirrer at a temperature of 110 °C and a speed of 200 rpm. Following a 24-h stirring period, the solution was evenly coated onto a glass plate. The plate was then left to stand for 12 h to allow for solvent removal. Subsequently, the plate was placed in a vacuum oven at 150 °C for 6 h to ensure complete solvent removal. The film was then separated from the glass plate using deionized water and finally placed in an oven at 60 °C to remove moisture. The thickness of the prepared COC film was measured at 9–11 μm.

Preparation of PEI film: The first step in the procedure was to dissolve 1.6 g of PEI in 10 mL of NMP. The mixture was placed on a magnetic stirrer and stirred at a temperature of 60 °C and a speed of 250 rpm. Following a 12-h stirring period, the solution was transferred to a vacuum oven for a duration of 2 h. This step was essential for the removal of bubbles that had been generated during the stirring process. The solution was then evenly coated onto a glass plate and placed in an oven for drying. The drying process was conducted at 60 °C for 4 h, 80 °C for 2 h, 120 °C for 2 h, 150 °C for 2 h, and 200 °C under vacuum for 2 h. The drying time was calculated from the moment the temperature inside the oven reached the preset temperature. This process ensured complete removal of the solvent. The subsequent steps were analogous to those for COC film preparation and will not be reiterated here. The thickness of the prepared PEI film was measured at 9–11 μm.

Self-healing performance test sample preparation: The film should be cut into rectangular samples measuring 4 × 1.5 cm. The aluminum electrodes, measuring 3 × 1 cm, were deposited on both sides of the film using a thermal evaporation coating machine. The electrode thickness was 100 nm, and the vacuum pressure during evaporation was below 5 × 10^−3^ Pa. It is imperative that the overlapping area between the upper and lower electrodes is precisely 1 × 1 cm. The prepared samples are displayed in Figure 1b.

### 2.3. Characterization and Measurements

The temperature resistance of the sample was tested using a differential scanning calorimeter (DSC, NETZSCH DSC 214, Selb, Germany) with a heating atmosphere of nitrogen (purity 99.999%) and a heating/cooling rate of 10 °C/min. The initial heating procedure served to erase the thermal history of the specimen, and the ensuing results were derived from the second heating curve. The utilization of a thermogravimetric analyzer (TG, HITACHI STA200, Tokyo, Japan) is imperative for the precise measurement of the change in sample mass with temperature. The self-healing occurrence frequency and capacitance changes of the sample were tested using the self-healing performance test platform shown in Figure 1c. The test temperatures were 80 °C, 120 °C, and 150 °C, with an applied electric field strength of 200 MV/m. The test duration was 16 h, with capacitance measurements taken at hourly intervals (data acquisition card: NI 6002 produced by National Instruments (Austin, TX, USA); LCR digital bridge: TH2826A produced by Changzhou Tonghui Electronic Co., Ltd., Changzhou, China). The microscopic structure information of the sample was obtained using a Fourier transform infrared spectrometer (FTIR, Thermo Fisher Scientific Nicolet iS20, Waltham, MA, USA). The bandgap width (Eg) of the sample was measured using ultraviolet–visible spectroscopy (UV–Vis, Shimadzu UV-3600i Plus, Fukuoka, Japan). The surface morphology of the self-healing site of the sample was observed using a SEM (Thermo Apreo 2S, Waltham, MA, USA). The elemental composition and content at the self-healing site were obtained using EDS (Oxford Ultim Max 40, Fukuoka, Japan). The energy storage tester (PolyK CPE1901, LLC, State College, PA USA) was utilized to assess the breakdown and energy storage performance of the sample at 150 °C. The test voltage rise rate was 200 V/s, and the energy storage test waveform was a triangular wave with a frequency of 100 Hz. The evaporation process during self-healing of the dielectric was simulated using Materials Studio (MS 2020) and Amsterdam Density Functional (ADF 2019) software. The temperature was set at a range of 7000 K to 300 K, the reaction time was set to 100 ps, the total number of steps was set to 400,000, and the step size was set to 0.25 fs [24].

## 3. Results

### 3.1. Thermal Properties

**Figure 2:**
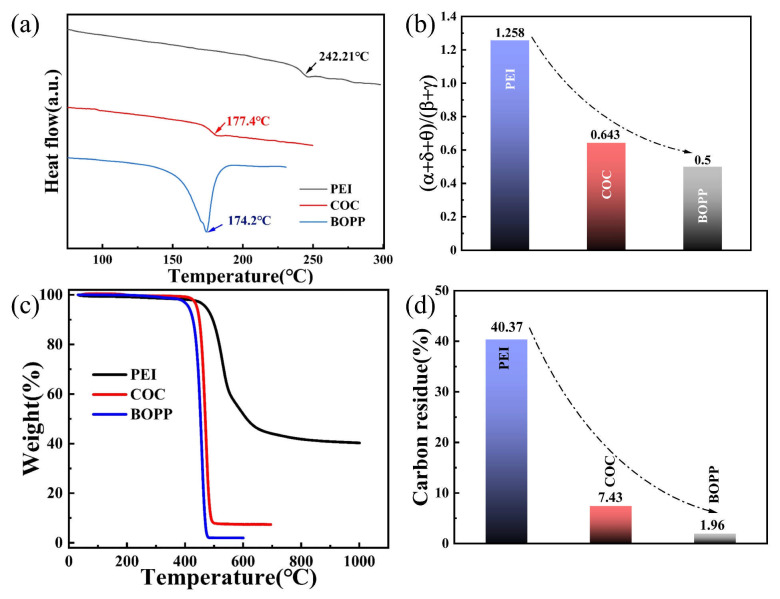
(**a**) DSC curves, (**b**) TG curves, (**c**) carbon-hydrogen ratio, and (**d**) (α + δ + θ)/(β + γ) of PEI, COC, and BOPP.

Figure 2a illustrates the DSC curves of PEI, COC, and BOPP. The endothermic peak of PEI was approximately 242.2 °C, corresponding to its T_g_. The endothermic peak of COC was approximately 177.4 °C, corresponding to its T_g_. BOPP exhibited the least favorable temperature resistance among the three materials, with an endothermic peak of approximately 174.2 °C, corresponding to its melting temperature. The (α + δ + θ)/(β + γ) for PEI was 1.258, while (α + δ + θ)/(β + γ) for COC and BOPP was only 0.643 and 0.5, respectively, as shown in Figure 2b. As demonstrated in Figure 2c, the temperatures at which the mass of PEI, COC, and BOPP decreased to 90% were 495.9, 448.4, and 317.3 °C, respectively, and the temperatures at which decomposition was complete were 906.7, 510.6, and 483.9 °C, respectively. The carbon residue content remaining after complete polymer decomposition was 40.37%, 7.43%, and 1.96%, respectively, as illustrated in Figure 2d. The aromatic structure in PEI is a rigid planar structure formed by sp^2^ hybridization between carbon atoms, which effectively restricts the rotation and movement of the molecular chain segments. Its delocalized π electron system makes it difficult for chemical bonds to break at high temperatures, resulting in high T_g_ and thermal decomposition temperatures for PEI [25,26]. However, due to the ring-shaped conjugated structure present in the aromatic structure, carbon atoms are required to be connected through unsaturated bonds. Consequently, the ratio of (α + δ + θ) to (β + γ) was elevated, leading to an increased quantity of carbon residue content following high-temperature thermal degradation. The conjugated structure of PEI is associated with inferior insulation performance in comparison to COC and BOPP. Additionally, the propensity for self-healing is increased, resulting in a more pronounced decrease in capacitance. Subsequent tests of self-healing and insulation performance will provide further insight into this phenomenon [22].

### 3.2. Self-Healing Properties

**Figure 3:**
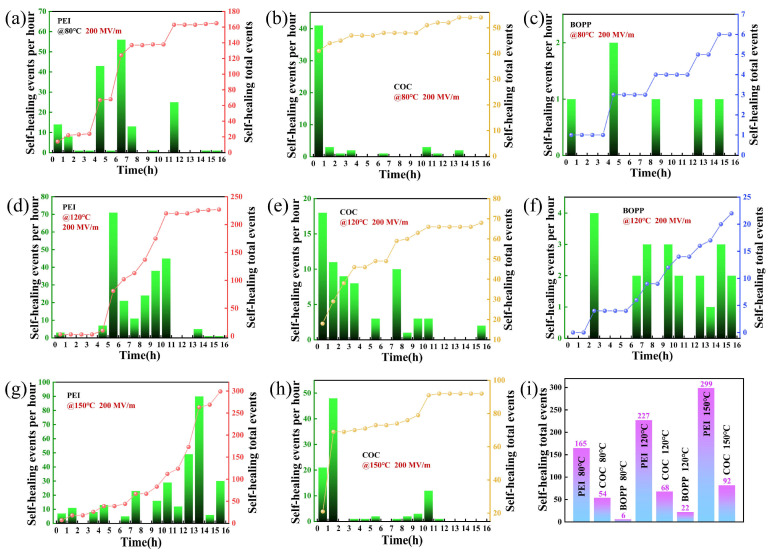
Dynamic changes in the number of self-healing events at 80 °C for (**a**) PEI, (**b**) COC, (**c**) BOPP; at 120 °C for (**d**) PEI, (**e**) COC, (**f**) BOPP; at 150 °C for (**g**) PEI, (**h**) COC; (**i**) total number of self-healing events for all tests.

Figure 3a–c illustrates the number of self-healing cycles for PEI, COC, and BOPP at a temperature of 80 °C. PEI exhibited a total of 165 self-healing events during the 16-h test period, with the highest number of events occurring between 5 and 7 h; COC demonstrated a total of 54 self-healing events, with more than 40 occurring within the first hour; and BOPP underwent a total of 6 self-healing events, which were distributed relatively evenly. As shown in Figure 3d–f, at a test temperature of 120 °C, PEI underwent a total of 227 self-healing events, with the vast majority occurring within 6–11 h; COC underwent a total of 68 self-healing events, mainly concentrated in the first 4 h; BOPP underwent a total of 22 self-healing events, with the most even distribution among the three materials. As demonstrated in Figure 3g,h, at a test temperature of 150 °C, PEI experienced a total of 299 self-healing events, predominantly distributed within 10–14 h; COC underwent a total of 92 self-healing events, primarily distributed within the first 2 h; Given that the melting point of BOPP is only 174.2 °C, when the test temperature reaches 150 °C, its use is precluded due to severe thermally induced curling. Figure 3i presents the total number of self-healing events for all tests. The total number of self-healing events for PEI was higher than that for COC and BOPP at all three test temperatures, primarily due to their molecular chain structures. As demonstrated in the FTIR spectrum depicted in Figure S1, the PEI molecular chain exhibited aromatic structures, resulting in a narrow E_g_, which facilitates carrier transport and impedes effective insulation [27]. As demonstrated in Figure S2, the E_g_ of PEI was 3.12 eV, that of COC was 5.32 eV, and that of BOPP was >6.5 eV. As the test time increased, the movement of internal charge carriers within PEI intensified, leading to a further decline in insulation performance. Consequently, the probability of self-healing increased with an increase in test time, and this trend became more pronounced at higher test temperatures. COC exhibited substandard mechanical properties and was susceptible to defects during preparation, which could readily induce self-healing. Therefore, COC underwent frequent self-healing in the early stages of testing [28,29]. COC and BOPP had wide Eg values, so COC rarely self-healed in the later stages of testing, and BOPP self-healed fairly evenly throughout the entire testing period.

**Figure 4:**
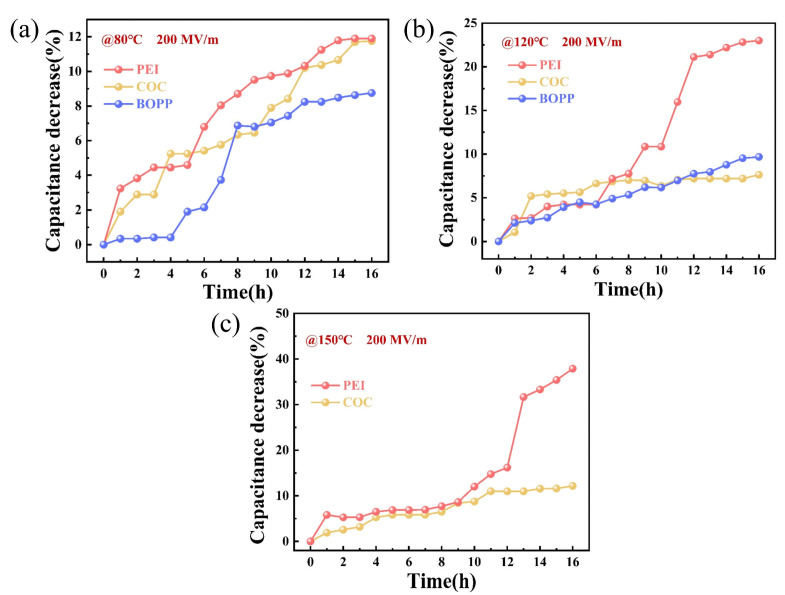
Capacitance decrease in (**a**) PEI (**b**) COC, and (**c**) BOPP.

In polymer film capacitors, capacitance is a pivotal metric for assessing the service life of the device. The process of self-healing, which is the dissolution of the metal electrodes deposited on both sides of the film, leads to the permanent impairment of the capacitance. Typically, when capacitance diminishes by 5–10%, the capacitor is considered to have failed [30,31]. The alterations in capacitance of the three materials during the 16-h test period are illustrated in Figure 4. After testing at 80 °C, the capacitance of PEI, COC, and BOPP decreased by 11.90%, 11.76%, and 8.75%, respectively; after testing at 120 °C, the capacitance decreased by 23.00%, 7.63%, and 9.67%, respectively; after testing at 150 °C, the capacitance of PEI and COC decreased by 37.88% and 12.18%, respectively. This temperature is close to the melting point of BOPP. Due to severe deformation of the sample during testing, the sample failed. The degree of capacitance decay of PEI increased significantly with rising temperature, while that of COC and BOPP showed no significant changes. Furthermore, COC displayed a substantially higher frequency of self-healing events in comparison to BOPP. However, the capacitance decay values for these two materials did not exhibit a significant disparity.

**Figure 5:**
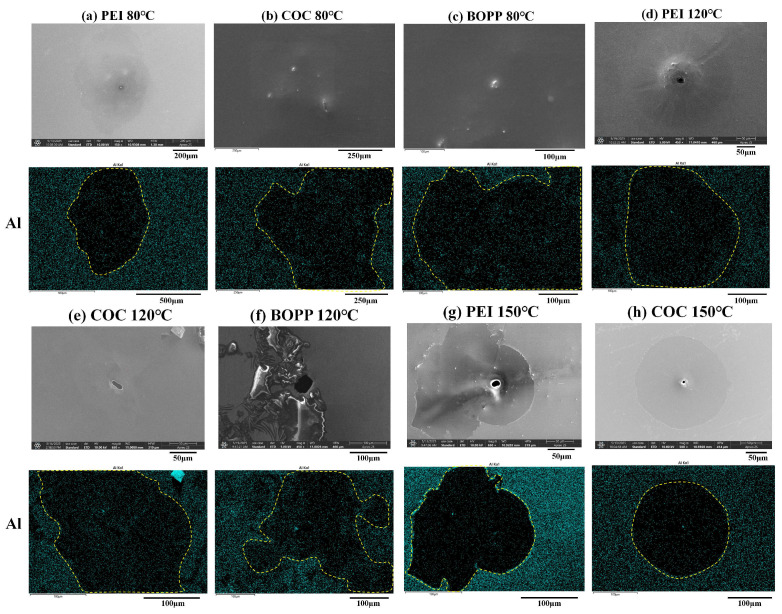
Microscopic morphology and Al element distribution map of self-healing sites.

In order to gain a more intuitive understanding of the impact of self-healing on capacitors, this study used SEM and EDS to observe the self-healing sites produced by three types of dielectric materials after testing at 80, 120, and 150 °C. The results are shown in Figure 5 and Figure S3. When self-healing occurs, the arc released by dielectric breakdown causes the local temperature to rise rapidly, evaporating the surrounding dielectric [32,33]. This formed the black holes shown in the center of the SEM images in Figure 5d–h. As illustrated in Figure 5a–c, the self-healing holes formed under the test conditions at 80 °C were not readily apparent. This indicates that high temperatures reduce the accumulation of conductive deposits at self-healing sites. The dielectric evaporation process is accompanied by the formation of a substantial amount of high-temperature gas, which diffuses outward, clearing the surrounding electrodes and exposing the dielectric, causing the sample to reform insulation [34]. Consequently, as illustrated in Figure 5 and Figure S3, the EDS image revealed a decrease in aluminum content and an increase in oxygen content within the area where the electrode has been removed. Additionally, the presence of silicon elements in the image is attributable to the utilization of silicone oil during the self-healing test. This oil exhibits a high affinity for organic matter and a low tendency to be removed. The image revealed a sudden decrease in carbon content at a specific location, accompanied by a concomitant increase in silicon content within the silicon element image. Consequently, this study determined the cleaning area of the electrode based on the alterations in aluminum elements within the energy spectrum and marks it in the image. As the temperature rose, the area of electrode removal after self-healing decreased for PEI, COC, and BOPP [13]. This indicates that, the higher the temperature to which PEI, COC, and BOPP are subjected, the lower the extent of damage to the electrodes during a single self-healing event.

### 3.3. Insulation Performance

**Figure 6:**
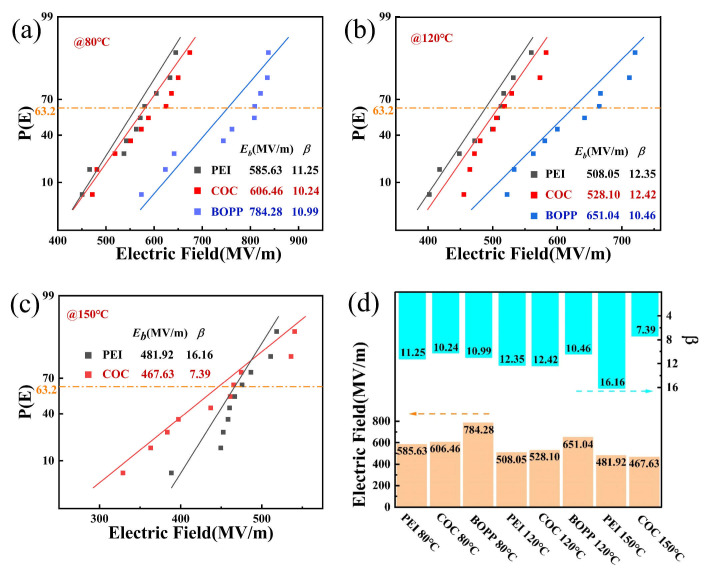
Breakdown performance of (**a**) PEI, (**b**) COC, (**c**) BOPP; (**d**) the comprehensive summary of E_b_ and β for all the samples.

In order to observe the effects of self-healing on the electrical properties of the three polymers, this study conducted breakdown tests on film samples coated with 3 mm diameter aluminum electrodes at different temperatures. The two-parameter Weibull analysis was employed to investigate the breakdown strengths of dielectrics and can be described by P(E) = 1 − exp(−(E/E_b_)^β^), where E is the measured breakdown strength, E_b_ is the characteristic breakdown strength at which the probability of the dielectric breakdown is 63.2%, P(E) is the cumulative probability of electric failure, and β is a parameter related to the reliability of the dielectrics [35]. Figure 6a depicts the cumulative failure probability of the three samples at 80 °C. The arrangement of molecular chains within BOPP, which exhibit an orderly disposition, result in the formation of crystalline regions in specific domains. The strong intermolecular forces and reduced free volume impede the mobility of molecular chains and charges, thereby conferring markedly enhanced insulation properties in comparison to those of COC and PEI. Figure 6b shows the cumulative failure probability of the three samples at 120 °C. The inadequate temperature resistance of BOPP results in a substantial intensification of molecular chain movement and a significant enhancement of charge mobility. These phenomena resulted in the most significant decline in insulation performance and the poorest stability of sample quality. Figure 6c shows P(E) of the three samples at 150 °C. The E_b_ values for COC are similar to those for PEI, but the sample stability of COC is significantly lower than that of PEI. Figure 6d provides a comprehensive summary of E_b_ and β for all the samples. It has been demonstrated that the band gap of COC is higher than that of PEI. This higher band gap results in stronger restrictions on carrier movement. However, its T_g_ is lower than that of PEI, resulting in greater molecular chain mobility and a larger free volume, which accelerates carrier movement [27]. Consequently, at temperatures of 80 °C and 120 °C, COC demonstrates marginally higher E_b_ in comparison to PEI [27]. Conversely, at 150 °C, COC exhibits slightly lower E_b_ than PEI.

## 4. Discussion

**Figure 7:**
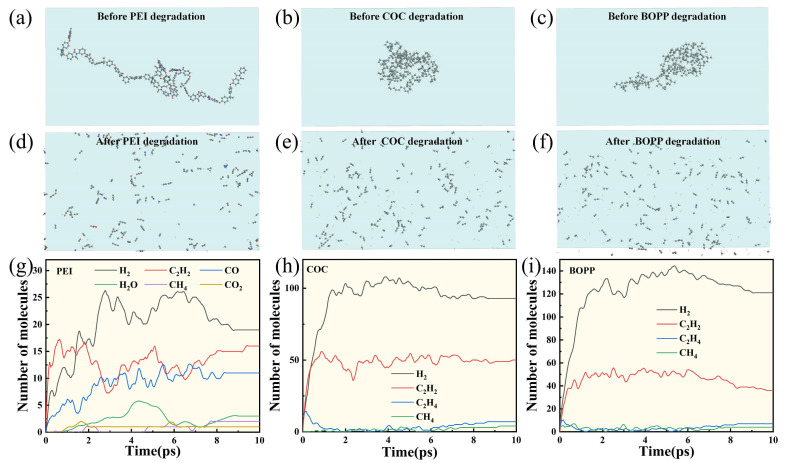
PEI, COC, and BOPP molecular chain (**a**–**c**) models before degradation; (**d**–**f**) models after degradation; (**g**–**i**) gas products after degradation.

In order to better explain the self-healing performance test results, this study employed MS to construct the molecular chains of the three polymers, selected the forcite module for structural optimization, and established the models shown in Figure 7a–c. Subsequently, the models were imported into ADF, and the ReaxFF reaction force field was employed to simulate the process from self-healing to stabilization in the dielectric materials [24]. As illustrated in Figure 7d,f, the model of the dielectric underwent decomposition into smaller molecules upon completion of the simulation. Figure 7g illustrates the variation in the yield of gas products from PEI, predominantly comprising H_2_, C_2_H_2_, CO, H_2_O, CH_4_, and CO_2_. Among these, H_2_, C_2_H_2_, and CO had the highest concentrations, with quantities of 19, 16, and 11, respectively. Figure 7h shows the changes in the quantities of gas products from COC, with H_2_ and C_2_H_2_ being the most abundant, at 93 and 50, respectively. As illustrated in Figure 7i, there was the composition of gas products from COC, with H_2_ and C_2_H_2_ emerging as the most prevalent, with quantities of 121 and 36, respectively. The specific heat capacity of hydrogen is 14.3 J/(g·°C), which is significantly higher than that of other gases in the gas products. Furthermore, hydrogen has a relatively weak destructive effect on electrodes. COC and BOPP have been observed to generate a substantial amount of gas during the process of self-healing, and when undergoing expansion, they will affect an increased area of the electrode. However, given that the gas produced is primarily hydrogen, the area of the electrode damage retains a significant quantity of electrode material. Consequently, at temperatures of 80 and 120 °C, the boundary of the area where the electrode was removed was not readily discernible. However, since hydrogen accounted for a smaller proportion of the gas products in PEI, the boundary of the cleared electrode area was clearly defined. As the test temperature increased, the dielectric insulation performance decreased, and the number of self-healing events increased. However, in capacitance testing, the capacitance of COC and BOPP did not undergo significant changes with increasing temperature. This phenomenon may be attributed to the fact that, at lower temperatures, the boundaries between the gas products of COC and BOPP and the cleared areas of the electrodes are not clearly defined. As test time progressed, the degree of damage to these boundary areas gradually increased, resulting in a continuous decline in overall capacitance. As the temperature increased, the boundary of the cleared area of the electrode became more clearly defined, and the extent to which the boundary area caused a continuous decrease in overall capacitance became less significant. Consequently, the degree of capacitance decrease in COC and BOPP remained relatively constant. This study is the first to explore changes in self-healing properties from the perspectives of materials and temperature, innovatively using microstructural characterization and macro-performance testing to reveal the relationship between the number of self-healing events, electrode morphology, and capacitance during the self-healing process.

## 5. Conclusions

In summary, the present study selected PEI, COC, and BOPP—three dielectric materials with different temperature resistance—as research objects and systematically tested their thermal properties, self-healing properties, and electrical properties at 80, 120, and 150 °C. The following conclusions were derived from the aforementioned data:

(1) Under test conditions with an applied electric field of 200 MV/m, as the test temperature increased, the capacitance of PEI decreased by 11.90%, 23.00%, and 37.88%, respectively; that of COC decreased by 11.76%, 7.63%, and 12.18%, respectively; and that of BOPP decreased by 8.75% and 9.67%, respectively.
(2) The gas products formed by COC and BOPP were far more abundant than those formed by PEI, and hydrogen accounted for a higher proportion. When self-healing occurred at 80 and 120 °C, the boundaries of the cleared areas on their electrodes were not easily discernible.
(3) As the test temperature increased, the total number of self-healing events for COC and BOPP increased, the damage to the electrodes caused by a single self-healing event decreased, and the capacitance remained largely unchanged.

## Data Availability

The data presented in this study are available on request from the corresponding author due to data privacy principles.

## Supplementary Materials

The following supporting information can be downloaded at: https://www.mdpi.com/article/10.3390/ma18174033/s1, Figure S1: FTIR spectra of PEI, COC, and BOPP; Figure S2: UV–Vis spectra of PEI, COC, and BOPP; Figure S3: Distribution of O and Si elements at the self-healing site.
