# Supplementary material for: Study on the Effect of Temperature on the Self-Healing Behavior of Film Capacitor Dielectrics
Source: Materials (Basel). 2025 Aug 28;18(17):4033. doi: 10.3390/ma18174033 (PMC12429509; doi:10.3390/ma18174033)
Supplement: Supplementary file 1 [file materials-18-04033-s001.zip › materials-3825416-supplementary.pdf]

Supporting Information

Study on the Effect of Temperature on the Self-healing Behavior of Film Capacitor Dielectrics

Mengjia Feng[1,2,3,4,*], Zhiguo Jia[1,2], Yancheng Liu[1,2], Yandong Liu[1,2], Jia Shi[1,2], Chaoyue Zhao[1,2], Tianqi Sun[1,2], Hongbo Liu[1,2] and Yunqi Xing[1,2]

1 State Key Laboratory of Intelligent Power Distribution Equipment and System, Hebei University of Technology, Tianjin 300123, China; 202321401044@stu.hebut.edu.cn (Z.J.); 202321401053@stu.hebut.edu.cn (Y.L.); 202421401116@stu.hebut.edu.cn (Y.L.); 202431402155@stu.hebut.edu.cn (J.S.); 202431402163@stu.hebut.edu.cn (C.Z.); 202421401132@stu.hebut.edu.cn (T.S.); hbliu@hebut.edu.cn (H.L.); yqxing@hebut.edu.cn (Y.X.)

2 School of Electrical Engineering, Hebei University of Technology, Tianjin 300123, China

3 National Key Laboratory of Electronic Thin Films and Integrated Devices, University of Electronic Science and Technology of China, Chengdu, 611731, China

4 Key Laboratory of Engineering Dielectrics and Its Application, Ministry of Education, Harbin University of Science and Technology, Harbin 150080, China

* Correspondence: mjfeng@hebut.edu.cn

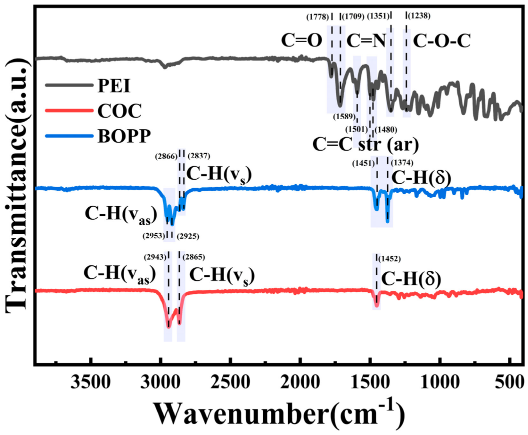

Figure S1. FTIR spectra of PEI, COC, and BOPP.

Figure S1 presents the FTIR spectra of PEI, COC, and BOPP. The PEI spectrum exhibits characteristic peaks at 1589, 1501, and 1480 cm^{-1} corresponding to aromatic C=C stretching vibrations (abbreviated as C=C str [ar]), indicating the presence of aromatic structures in the PEI molecular chain. Additionally, characteristic peaks are observed at 1778 cm^{-1} (C=O stretching vibration in the imide ring), 1709 cm^{-1} (asymmetric C=O stretching vibration in the imide ring), 1351 cm^{-1} (C-N stretching vibration), and 1238 cm^{-1} (C-O-C stretching vibration). The COC spectrum shows characteristic peaks at 2953 and 2925 cm^{-1} (asymmetric CH stretching, abbreviated as C-H [ν_{as}]), 2866 and 2837 cm^{-1} (symmetric CH stretching, abbreviated as C-H [ν_s]), and 1451 and 1374 cm^{-1} (in-plane CH bending, abbreviated as C–H [δ]). The BOPP spectrum displays characteristic peaks at 2943 cm^{-1} (C-H [ν_{as}]), 2865 cm^{-1} (C-H [ν_s]), and 1452 cm^{-1} (C–H [δ]).

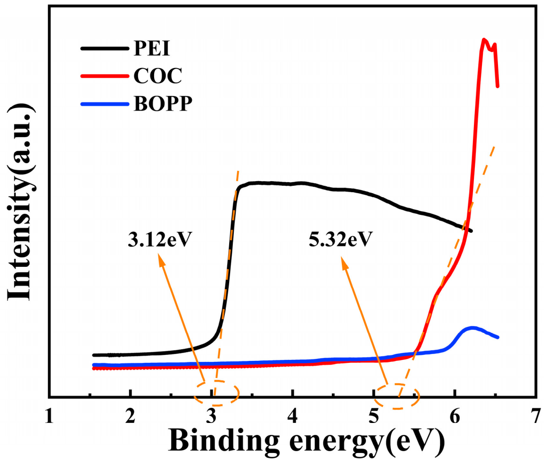

Figure S2. UV–Vis spectra of PEI, COC, and BOPP.

Figure S2 shows the UV–Vis spectra of PEI, COC, and BOPP. E_g of PEI is 3.12 eV, while that of COC is 5.32 eV. In contrast, BOPP exhibits significant E_g exceeding 6.5 eV.

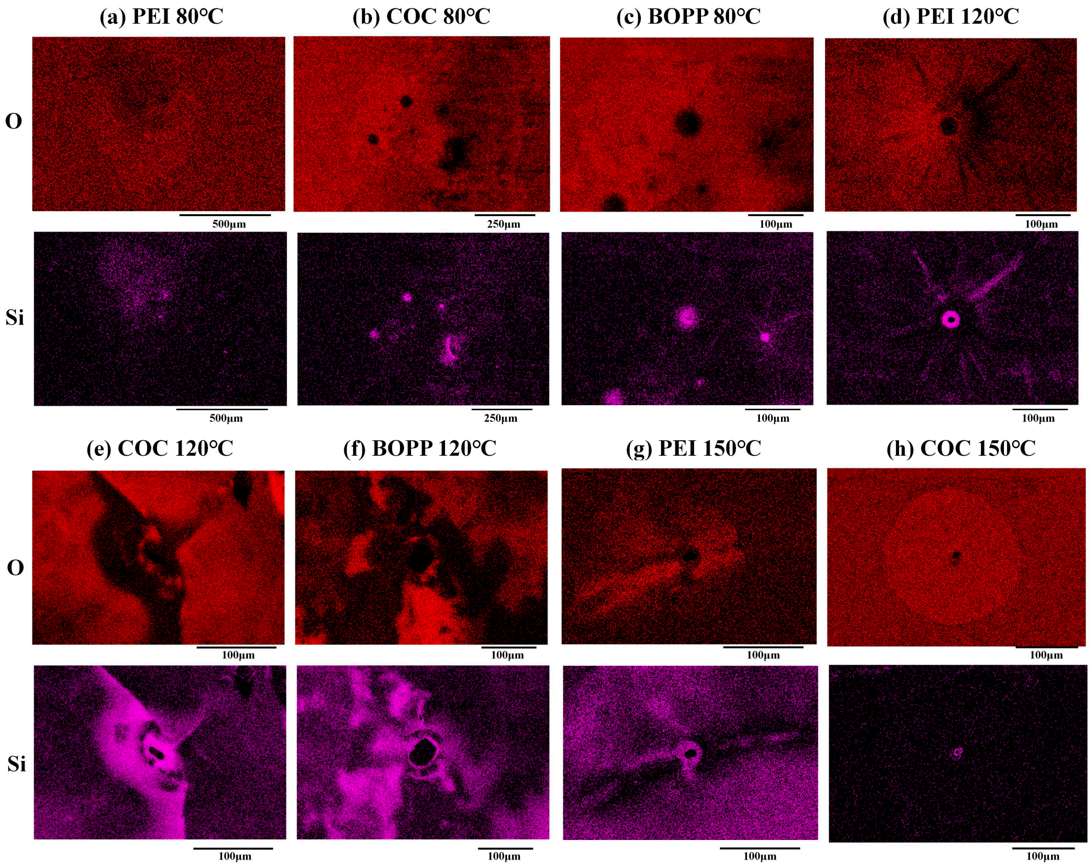

Figure S3. Distribution of O and Si elements at the self-healing sites.
